# Effect of Antibiotic Compared to Non-Antibiotic Dry Cow Treatment on the Bacteriological Cure of Intramammary Infections during the Dry Period—A Retrospective Cross-Sectional Study

**DOI:** 10.3390/antibiotics12030429

**Published:** 2023-02-21

**Authors:** Stephanie Müller, Julia Nitz, Anne Tellen, Doris Klocke, Volker Krömker

**Affiliations:** 1 Faculty II, Microbiology, Hannover University of Applied Sciences and Arts, Heisterbergallee 10a, 30453 Hannover, Germany; 2 Faculty of Health and Medical Sciences, Department of Veterinary and Animal Sciences, Section for Production, Nutrition and Health, University of Copenhagen, Grønnegårdsvej 2, 1870 Frederiksberg, Denmark

**Keywords:** dairy cows, antimicrobials, selective dry cow treatment, teat sealant, udder health, subclinical mastitis

## Abstract

Antimicrobials are widely used to cure intramammary infections (IMI) in dairy cows during the dry period (DP). Nevertheless, the IMI cure is influenced by many factors and not all quarters benefit from antimicrobial dry cow treatment (DCT). To evaluate the true effect of antibiotic DCT compared to self-cure and the role of causative pathogens on the IMI cure, a retrospective cross-sectional study was performed. The analysis included 2987 quarters infected at dry-off (DO). Information on DCT, causative pathogens, somatic cell count, milk yield, amount of lactation, Body Condition Score, and season and year of DO were combined into categorical variables. A generalized linear mixed model with a random cow, farm and year effect and the binary outcome of bacteriological cure of IMI during the DP was conducted. In the final model, a significant effect (*p* < 0.05) on DP cure was seen for the DO season and the category of causative pathogens (categories being: *Staphylococcus aureus*, non-aureus staphylococci, streptococci, coliforms, ‘other Gram-negative bacteria’, ‘other Gram positive bacteria’, non-bacterial infections and mixed infections), while antibiotic DCT (vs. non-antibiotic DCT) only showed a significant effect in combination with the pathogen categories streptococci and ‘other Gram-positive bacteria’.

## 1. Introduction

Clinical mastitis (CM) is one of the most important diseases in dairy cows when considering ecological aspects [1,2], animal health, and antimicrobial usage (AMU) [1,3,4]. The dry period (DP) plays a crucial role in the udder health of dairy cows, as it gives the udder tissue a chance to regenerate and cure infections [4,5,6]. Nevertheless, there is also a high risk of new intramammary infections (IMI) occurring during the DP, especially at the beginning, when the keratin plug has not yet formed and shortly before calving with the start of the new secretion [5]. To achieve high cure rates and a low number of new IMI, blanked dry cow treatment (BDCT) has been an often used and recommended practice over the last decades. However, the European Union (EU) Regulation 2019/6 [7] forbids the prophylactic use of antibiotics (AB) as routine measurements in veterinary medicine. Thus, routine BDCT where every cow is treated with dry cow AB at dry-off (DO) is banned in the EU, with the regulation entering into force in 2022. With selective dry cow treatment (SDCT) being the alternative, new challenges occur. The aim is to only treat cows that will benefit from antimicrobial treatment with AB at DO, while the other animals remain untreated or are administered only a non-AB internal teat sealant (ITS). However, since mastitis is a multifactorial disease, there are no exact guidelines on how to find those animals or quarters that would benefit from AB treatment at DO. Protection against new IMI can be achieved with ITS [8,9,10,11], and the preventive effect of dry cow AB declines during the DP due to decreasing levels of the active constituents to levels below the minimal inhibition concentration [12]. This implies that antimicrobial dry cow treatment (DCT) is only necessary in infected cows to improve the chances of a cure. To detect those infected cows or quarters at DO, different approaches are eligible. Besides laboratory bacteriological culture, somatic cell count (SCC) thresholds [13,14,15], the California Mastitis Test [16,17], on-farm-culture tests [18,19] and others as well as combinations of these methods can be used. Additional risk factors for IMI cures can be included in this decision, such as the amount of lactation or the number of CM cases in the previous lactation [13]. There are many factors described as being associated with the cure rate during the DP, varying in their significance between studies. Regarding the influence of dry cow AB on the cure of IMI, BDCT is often compared to SDCT [20,21] but seldom to a non-treated control group [22]. Halasa et al. [22] presented an almost 1.8 times higher cure rate for SDCT and BDCT compared to generally non-treated cows. These results show, on one hand, a positive effect of AB on the chance of cure and, on the other hand, that SDCT and BDCT achieve the same cure rates under certain circumstances [22]. Several studies describe that when AMU at DO is reduced (e.g., by using SDCT instead of BDCT), this does not have to lead to a decline in udder health in early lactation [21] or long term [23,24]. However, most previous studies on SDCT suggest that each cow or quarter with a present infection at DO would benefit from AB treatment at DO, using either algorithm-guided protocols, cow-side tests, or culture-guided protocols to evaluate the infection status of cows or quarters at DO. To allow for a more precise prediction of whether AB treatment is needed, the true effect of AB DCT needs to be further investigated. By knowing how many quarters infected by different pathogens at DO benefit from AB DCT, a more precise decision making and a further reduction in AMU might be possible. Nevertheless, evaluable recent results on the additional effect of AB treatment compared to self-cure are scarce. In studies comparing SDCT with BDCT, self-cure of quarters is not always reported, or it is reported as a secondary result, with a low number of infected but non-treated quarters [25]. Moreover, considering the influence of dry cow AB on infections caused by different pathogens, pathogen-specific cure rates are needed within AB and non-AB treatment groups. Though pathogen-specific cure rates during the DP are reported in some studies, these also are often secondary objectives and are lacking in the number of cases for some pathogens, while others could not report pathogen-specific cure rates at all, as the IMI are often defined by SCC thresholds, or the cure rate is only described as the overall cure [13,26]. 

The objective of this retrospective cross-sectional study was to determine farm-, cow-, and quarter-level factors associated with DP cure. Furthermore, the aim is to estimate the true effect of AB DCT on the cure of existing IMI, dependent on the causative pathogen by comparing the DP cure of non-treated quarters with those treated with AB at DO for different pathogen categories. Knowledge of pathogen specific AB-cure and self-cure rates can improve decision making in SDCT and support evidence-based reduction of AMU. 

## 2. Results

### 2.1. Data Selection 

Eligible information for the analysis was found for 14,593 quarters in the primary dataset (17,288). Since only cow lactations with reported data on four quarters were considered, another 607 quarters (161 cows) were excluded. To adjust for DCT only, cows with CM, udder injuries, or other reported illnesses at DO were excluded to rule out DO unrelated AB treatment. Out of the remaining 13,326 quarters (of 2926 cows), homeopathic treated quarters (n = 83) were excluded to limit the treatment effect to AB DCT. Another 3039 quarters could not be used for the analysis due to contaminated milk samples. By including only milk samples taken within 5 days before DO as well as between calving and 19 days *post partum* (*p.p*.), with a DP of more than 14 days, 6503 quarters of 1745 cows on 99 dairy farms were left for the analysis. Out of these quarters, 454 were included for more than one lactation. In order to examine the effects on the IMI cure during the DP, only quarters infected at DO were eligible for the analysis. Thus, the final dataset consisted of 2987 quarters of 1295 cows from 96 farms, with 92 quarters being included for more than one lactation.

### 2.2. Descriptive Results 

The geometric mean of the SCC at DO was 546 × 10^3^ cells/mL overall, this being higher in the AB treated group (625 × 10^3^ cells/mL) than in the non-AB group (502 × 10^3^ cells/mL), although the median was lower for each SCC measurement. In comparison, geometric means for milk yield, Body Condition Score (BCS), parity, and DP length were similar in both groups (Table 1). 

Missing values were detected for SCC (2.1%), milk yield (2.3%), BCS (12.8%), and parity (6.0%). The predominant pathogens at DO were non-aureus staphylococci (NAS) with 1094 (36.63%) infected quarters, followed by the category ‘other Gram-positive bacteria’ with 668 (22.36%) infected quarters at DO (Table 2). Although there were overall more quarters dried off without AB than with AB, the proportional distribution of pathogens detected in DO milk samples was mostly consistent between pathogen categories. Only for coliforms were there slightly more quarters treated with AB, than without and the proportion of quarters treated without AB was noticeably higher for non-bacterial infections compared to other pathogen groups. Altogether, 2342 of the 2987 (78.41%) quarters were cured during the DP. The total cure rate in the AB treated group was 84.50% and was higher than in the non-AB group (75.06%), although the difference varied between the pathogen categories (Table 2). The lowest cure rate was observed for mixed infections (two different pathogen species found in the milk sample) for the AB cure as well as self-cure (66.67 and 63.32%), while streptococci showed the greatest difference between the cure rates, with 94.12% for the AB cure vs. 74.12% for self-cure (Table 2). ITS was administered to a total of 928 quarters (246 in the AB treated group, 682 in the non-AB group). Adequate information on the used product or active compound was missing in 91 cases for ITS and 11 cases for the antibiotic DCT. The most frequently used AB were medications with a simple penicillin (n = 601), followed by formulations with combined penicillin and aminoglycosides (n = 302), while only 144 quarters were treated with cephalosporins. Considering all 6503 quarters eligible for the analysis (including non-infected quarters), 30.9% (n = 2009) of the quarters were considered as new IMI (31% of non-AB and 30% AB treated quarters). 30.8% of these new IMI were caused by NAS, followed by 14.3% of new IMI by coryneforms and 15.3% by coliforms. *S. aureus* showed a similar new IMI rate as streptococci as a group (8.8% and 8.7%), while 11.3% of the new IMI were caused by two different pathogens that were not isolated at DO. 

### 2.3. Generalized Linear Mixed Model

Only the categories of causative pathogen, season of DO, DCT (AB vs. non-AB), and the combination of causative pathogen group and DCT were kept in the final model as fixed effects. However, by including the interaction of the pathogen with DCT in the model, DCT alone did not show a significant effect anymore, but was kept in the model due to being part of the interaction (Table 3). Farm, cow, and year of DO were included as random effects. Infections caused by *Staphylococcus (S.) aureus*, streptococci, coliforms and ‘other Gram-positive bacteria’ were associated with a higher chance of cure during the DP compared to mixed infections. AB treatment only showed a significant effect on DP cure in combination with some pathogens in the final model. More precisely, IMI caused by streptococci or ‘other Gram- positive bacteria’ presented a significantly higher chance of cure if treated with AB compared to non-AB treated quarters. Concerning the season of DO, cure was lower in cows dried off in spring (March until May) compared to cows dried off in the fall (September until November); see Table 3. Out of the included random effects, only the variables ‘cow’ and ‘farm’ showed a significant impact on DP cure. Estimated marginal means for the combination of treatment and causative pathogen are shown in Table 4. Taking all of the included variables into account, the predicted means for cure were 22.6 and 13.8% higher for streptococci and ‘other Gram-positive bacteria’ if treated with AB compared to self-cure (Table 4). 

## 3. Discussion

Since this is a retrospective cross-sectional study based on an existing dataset of previous studies, we had no detailed information on DCT protocols for each farm, and *post hoc* collection of missing data was not possible. Although quarter level information on DCT and infection status had to be complete, as the aim of the study was to evaluate the real effect of dry cow AB on the DP cure, quarters with missing information on other explanatory variables for the outcomes of cure (SCC, BCS, milk yield) were still included in the trial. This might have led to the premature exclusion of these variables compared to those with information given for all quarters and bias in our results. However, the number of quarters with missing information was mostly still small, around 2–6% of all quarters, and therefore should not lead to major variation. Only information on BCS at DO was missing for 13% of all quarters (Table 1).

### 3.1. Benefit of AB

The results of this study indicate that the effect of antibiotic DCT on the cure of IMI infections at DO, adjusted for the self-cure, has been overestimated by practitioners and farmers, considering that BDCT has been a widely recommended method for DCT over the last decades. Since AB treatment is meant to support the cure of bacterial infections, the cure rate in quarters treated with AB is expected to be higher than in untreated quarters, as described in previous studies [22,27]. Surprisingly, our results did not show a significant effect of AB treatment on the DP cure overall, although numerically, the cure rate was higher in AB treated cows (Table 2). Only in combination with the causative pathogen was a significant effect seen, with a 14–23% higher chance of cure for the two groups ‘other Gram-positive bacteria’ and *Streptococcus* spp. when treated with AB compared to the self-cure group.

This leads to the assumption that only quarters infected with certain Gram-positive bacteria benefit from AB treatment at DO. This could be partly explained by the mainly Gram-positive range of efficacy of most dry cow AB used in Germany. In this study, about 57% of the treated quarters received a simple penicillin-based product (cloxacillin, oxacillin, procaine penicillin). Furthermore, Bradley et al. [28] found coliforms, except *E. coli,* to be more likely to persist during the DP, even with AB DCT, while the self-cure rate for coliform infections was high (>93%) in our study. Since Gram-negative bacteria and NAS are often the cause of new IMI [28], an additional reason could be that a high percentage of the non-cured IMI were reinfections with the same pathogen species. Given that the protection of dry cow AB against new IMI decreases with the proceeding DP [12], new IMI, including reinfections, would appear in untreated quarters as well as in quarters treated with AB, especially at the end of the DP. However, the category of ‘other Gram-negative bacteria’, at 63 quarters, was the second smallest group after the non-bacterial infections. The coliforms category, on the other hand, was only slightly smaller than the category of *Streptococcus* spp. which showed a significant effect of AB DCT. Even though only a small number of cases were non-bacterial infections, these were grouped into an extra category so the outcome for the other categories would not be affected, since no effect of AB treatment was expected for this category. 

As the cure rate in AB treated quarters was about 9% higher than in non-treated quarters, the true benefit of AB (in addition to the self-cure) seems to be around 9% in infected quarters, dependent on the causative pathogen. Given that only 2987 quarters out of a total of 6503 that matched all of the criteria were infected at DO and hence included in the analysis, the absolute AB effect would be even smaller for the whole population with BDCT. 

### 3.2. Outcome Cure

The overall cure rate of 78.4% was high in our study, with higher cure rates in the AB and non-AB group compared to the findings of Halasa et al. [22], who reported a cure rate of 78% for BDCT and 46% for spontaneous cure although the AB cure rate for *Streptococcus* spp. was similar. Gundelach et al. [29] reported a bacterial cure for AB treated quarters of 86%, which is in line with our findings. The differences might be due to farm individual effects and differences in the prevalence of major mastitis pathogens. In our study, the predominant pathogens found in DO samples were NAS, as described in previous studies [30,31,32], while others found *Corynebacterium* spp. as the predominant pathogen species, followed by NAS at DO [28,29]. So far, non-AB cure rates considering pathogen-specific cure have rarely been presented. While Cameron et al. [25] found no differences in species-specific cure rates between treatment groups (BDCT vs. SDCT), our model indicates differences between pathogen groups regarding the chance of cure. The self-cure percentage of infections caused by NAS in our study was lower than the cure with AB treatment (68% and 78% respectively) as described in previous studies [27,28]. Although the cure rates described by Bradley et al. [28] were lower for NAS in different treatment groups, Vasquez et al. [27] showed higher cure rates for NAS in both treatment groups (77% in non-AB and 91% in AB, respectively) compared to our findings. Higher cure rates for NAS are also described by other previous studies [25,32]. Regarding *S. aureus* infections, the true cure rate might be lower, because of possible false-negative samples at calving due to intermittent shedding of *S. aureus*, although single quarter samples are reported to be sufficient to identify *S. aureus* IMI [33].

The season of DO had a significant influence on the DP outcome, which is consistent with the findings of Dingwell et al. [34]. Many factors might cause a seasonal influence on the DP outcome, such as increased heat stress [35] or a high prevalence of mastitis-causing pathogens, especially environmental pathogens in warm seasons [36], as well as the housing system and management of dry cows during the year (e.g., dry cows housed in barns during the fall and winter and in the field during spring and summer) [37]. Thus, cows dried off in spring or summer and likely to calve in warm months might be more exposed to environmental pathogens, which could lead to a lower chance of cure for quarters dried off in spring (March–May) compared to the fall (September–November), as found in the present study. Considering that multiple factors that could have had an influence on the season effect were not reported in this study, more research would be needed on this topic to evaluate the exact reason for the seasonal effect. To rule out special annual (environmental) effects, the year of DO was included as a random effect because the analysis included data from a total of four years. However, there was no significant effect of this included random effect seen in the final model. 

An effect of the farm on the DP outcome can be explained by many farm-individual factors. Predominant pathogens, barn and pasture management, as well as DCT protocols are some factors that can vary between farms and influence the DP cure [28,30,37]. In addition, the cow breeds were not the same on all farms included in this study, which might have an additional influence on the DP cure. We did not have detailed information on each farm, e.g., regarding prevalence of mastitis pathogens, bulk tank SCC, or management aspects, so we were unable to determine the reason for the farm effect more precisely in our research. The influence of the individual cow, included as a random effect, was hardly surprising, since each cow is different (age, genetics, mastitis background, immune system, and many other criteria). Previous studies report the SCC at DO, a background of clinical mastitis in the previous lactation, or the number of infected quarters at DO to have an influence on the DP cure [38,39]. This could, among other factors, be part of the effect the individual cow had on the DP in this study. Previous studies also reported a significantly higher chance of cure in the forequarters compared to the hindquarters [38,39,40]. However, this effect was not significant during the variable selection process and therefore not further investigated in the final model. Not significant, and thus not included in the final model, were the SCC, the milk yield and the number of lactations at DO, which is in line with the findings of Rowe et al. [41]. On the contrary, other studies reported a decreased chance of cure for multiparous cows or cows with a high SCC at DO [34,38,39,42]. The variations could be due to differences in the prevalence of major and minor pathogens as well as variations in the classification of the variable. Nonetheless, while previous studies often used a SCC threshold as an inclusion criterion, the mean SCC in our dataset was high but still connected with overall high cure rates. Another not significant variable in our model was the milk yield at DO, of which in previous studies the effect on *p.p* udder health is associated with an increased risk of new IMI due to an insufficient closure of the teat canal in high yielding cows [34,42], not a decreased DP cure. Despite our observation, BCS is being associated with the DP cure in the form of a decreased chance of cure in cows with a high difference in BCS during the DP [30].

As previously described by Dingwell et al. [40], in our results, the DP length had no significant influence on the DP cure, even though both very short and very long DP were included. Given that the main effect of dry cow AB is expected to take place at the very beginning of the DP, and the mean and median DP length were comparable for the AB and non-AB group, a great impact on the main outcome was not expected. 

The application of ITS also did not show a significant effect on the DP cure in our model. This was expected, since the main function of ITS is to protect the udder from new IMI by forming a physical barrier for pathogens to enter the teat canal, not to cure IMI [8,11]. However, there are also studies describing a positive effect of ITS on the DP cure [13,43]. Additionally, the protection from reinfection might have influenced our outcome. Nonetheless, since ITS were given to quarters in the AB and the non-AB group (23% and 35%, respectively), the impact on the outcome of cure (AB vs. non-AB) is supposed to be minor. To account for the various AB products used for DCT, a reduced model was built, although no significant effect of the different active compounds was found, which is in line with McMullen et al. [26], who did not find a clear difference in efficiency in curing IMI during the DP for different AB products. Contrary to our findings, other studies found variations in cure rates between some formulations [29,40]. To evaluate the effect of the DP cure for individual AB products, further research is needed.

### 3.3. Limitations and Significance of the Study

Since the dataset was compiled from two previous studies, the samples used for this analysis were not recent Despite the fact that some were taken more than ten years ago, this should not lead to a major bias in our results. For one thing, the distribution of pathogens found at DO in our research is comparable with the results of a study conducted in Germany more recently [31]. Furthermore, the active compounds in the AB products mainly used for DCT on the farms in this study are the same as in AB products still used for DCT in Germany. In addition, there is no indication of an increase in antimicrobial resistance in mastitis pathogens for the used AB substances over the last years [44,45]. Considering that the two previous studies took place in different years (one in 2014, one from 2008 until 2010), the cows and farms were not equally distributed over the years, which might lead to some bias. Furthermore, we neither took milk samples within the DP nor did we compare pathogen strains of the DP with stains of the calving samples. Hence, we could not separate persistent infections from reinfections with the same pathogen species. Furthermore, by using molecular diagnostic methods and identifying bacterial strains, more precise decisions could have been made regarding cure, new infection and persistent IMI. Thus, evaluating the cure only at species or even at the species-group level might lead to some bias in our results. In this study, IMI causing pathogens were determined using laboratory microbiological diagnostics regularly used for milk samples, and pathogens were determined at the species level or as a group of species (e.g., NAS, *Coryneforme* spp., ‘other streptococci’ or ‘other coliforms’). However, not identifying pathogens at a species or even a strain level only has an impact on differentiating persistent from cured and re- or new infected quarters. Therefore, any IMI evaluated as persistent in this study might actually be a reinfection with the same pathogen or a new infection with another strain of the same group, indicating a higher true-cure rate than found here. For most strain-groups, however, only up to 8% of the IMI were regarded as persistent. The highest proportion of not cured IMI were found for *Coryneforme* spp. and NAS higher (13.8% and 28.2%), so the greatest bias would be expected for the outcome for these pathogens. Nevertheless, like in our study, NAS are not generally identified at the species level. This goes in line with previous studies investigating the DP cure of IMI, also using NAS as one pathogen category, without further separation [13,22,27,31]. While recent studies investigate the prevalence and role of the different species of NAS (e.g., *S. chromogenes, S. epidermidis, S. haemolyticus*) as mastitis pathogens [41,46,47], and the risk of persistent IMI is described to vary between NAS species [46], single DP cure rates are not generally given. Considering the new IMI rate for NAS found in this investigation (31% of all new IMI), the amount of cured and reinfected quarters is expected to be high for this pathogen group, which should be kept in mind when interpreting our results. In order to take reinfections into account, further research is needed where the pathogen strains in DO and calving samples are determined. If the cure rates were adjusted for reinfections, the true cure rates would be even higher, especially for environmental pathogens, because more than 88% of the IMI found at calving are new IMI received during the DP or even *p.p*. before the *p.p.* sample was taken [28,31]. However, the influence of misinterpreted cures or persistent IMI, due to reinfections should be comparable for the AB and non-AB treated quarters, since most reinfections occur at the end of the DP or even after calving, when the effects of the AB given at DO have worn off. Overall, IMI at calving being misinterpreted as persistent IMI led to an underestimated cure rate. Likewise, the difference in cure between AB and non-AB treated quarters might differ, if the pathogen species are unequal between both groups. Nevertheless, this is unlikely for a study this size, and the proportion of overall new IMI are equal in both groups. Finally, all farms included in this study were organic farms. Results might differ for conventional farms since there are potential differences in the prevalence of certain pathogens, antimicrobial resistance, treatment protocols or management, and others.

Although many studies have investigated the influence of different cow or farm level factors or the difference between SDCT and BDCT on the DP outcome, the effect of AB DCT adjusted for self-cure is rarely reported for different pathogens on a large number of quarters. In order to account for the specific influence of different dry cow products or the pathogens on a species level, more research is needed. Nevertheless, the findings of this cross-sectional study indicate that the absolute beneficial effect of AB DCT is mainly seen in some Gram-positive pathogens, while no significant benefit is expected for infections with NAS or Gram-negative bacteria. This can support future decision-making for SDCT and encourage farmers and veterinarians to further reduce AMU if the farm and herd conditions allow it. Future studies might investigate DCT protocols that include pathogen-based treatment decisions. 

## 4. Materials and Methods

### 4.1. Data

The initial dataset used for this study was compiled from data originally collected for two previous studies that were conducted on organic dairy farms in Germany [13,48]. Considering quarters with information on several lactations as separate quarters, the initial dataset presented information on a total of 17,288 quarters from 3710 cows, with information on SCC at DO, microbiological diagnostics at DO and calving, milk yield at DO, DCT, number of lactations, BCS, and date of DO and calving gathered from 106 German farms. The cows were dried off between 2008 and 2010 and in 2014. Besides German Holstein cows, data had also been collected from Swiss Brown and Simmental cows in one of the studies [48]. Quarter milk samples had been taken at DO and shortly after calving. The primary dataset was examined and reduced to a smaller dataset including all information needed for the analysis. Quarter-level data were eligible for the analysis if there was complete information on farm and cow-ID, quarter position, DCT, IMI status at DO and calving, as well as the sampling date for the DO and calving samples for the respective lactation. Furthermore, the given data was scanned for information that could not be included in the analysis like contaminated milk samples or factors that might lead to bias in our results, like DO unrelated treatments. 

Farm individual information such as herd size, bulk tank SCC, and specific DCT protocols was not given for all farms in the dataset and hence not included in the analysis. Reported AB used for DCT on these farms are shown in Table 5, although not all products are licensed for DCT in Germany anymore. Regarding the use of ITS, Noroseal (2.6 g, Elanco Animal Health, Bad Homburg, Germany) and Orbeseal (2.6 g, Zoetis Deutschland GmbH, Berlin, Germany) were the reported products used on the farms in this study, although in some cases, the name of the product was not given. 

The cytomicrobiological diagnostics of quarter milk samples taken at DO and at calving had been performed in the laboratory of Hannover University of Applied Sciences and Arts, Hannover, Germany, in accordance with the guidelines of the German Veterinary Association [49]. Bacterial isolation for each sample was performed as routine microbiological diagnostic for milk samples and pathogens were determined at species level, genus level or species group, dependent on the bacteria, although bigger pathogen groups were built for the statistical analysis. The dataset contained quarter level information on SCC at DO, microbiological diagnostics at DO and calving, milk yield at DO, DCT, number of lactations, BCS and date of DO and calving.

### 4.2. Definitions

In this analysis, only bacteriological cure was examined. IMI cure was evaluated for each single udder quarter, by comparing the isolated pathogens of the DO and calving quarter milk samples. The IMI cure was defined as quarters that showed one or two pathogens in the DO sample and were either bacteriologically negative in the calving sample or infected with one or two pathogens other than those in the DO sample. Quarters that had at least one similar pathogen in the DO and the calving sample were considered as not cured, even if one of the two pathogens (if mixed at DO) was not found in the calving sample anymore. Pathogens found in the calving sample that were not isolated in the DO sample were regarded as new IMI. Samples with two different pathogens were considered as mixed infections, while quarter milk samples with more than two pathogens were classified as contaminated. IMI cure or new IMI were evaluated at the species level or, if the pathogen species was not determined, on the genus level or as a species group (e.g., NAS). For example, a quarter infected with *E. coli* at DO and *S. aureus* at calving was considered as cured and new infected, while an infection with *E. coli* at DO and calving was considered as not cured and not new infected, hence persistent (evaluation at species level). A quarter infected with NAS at DO and at calving was also considered as persistent (evaluation as a species group, pathogen species not exactly determined).

### 4.3. Statistical Analysis

To perform the dataset analysis, Microsoft Excel (2016) and SPSS 28.0, Chicago IL, USA were used, with the udder quarter as the statistical unit. For the binary outcome of IMI cure, independent variables were tested for statistical significance (*p* ≤ 0.20) using univariable analysis. Variables with *p* ≤ 0.20 were kept for the final multivariable model. Afterwards, statistically significant variables were included in a generalized linear mixed model (GLMM) with logit link function. 

The variables farm, cow, quarter, and year of DO were considered as random effects. The main explanatory variables were DCT (antibiotic or non-antibiotic treatment) and the causative pathogen. Since some pathogens were only isolated in a few milk samples, pathogen categories were built for the statistical analysis:

1. *S. aureus*;
2. NAS;
3. *Streptococcus (Sc.)* spp. including *Sc. uberis*, *Sc. dysgalactiae*, *Sc. canis* and other not further specified streptococcal bacteria;
4. coliforms, including *Escherichia coli*, *Klebsiella* spp., *Serratia marcescens*, and other not further specified coliforms;
5. other Gram-negative bacteria, including *Aeromonas*, *Pseudomonas*, *Proteus*, *Alcaligenes*, *Bordetella*, *Moraxella*, and *Acinetobacter* spp.;
6. other Gram-positive bacteria, including *Trueperella pyogenes*, *Corynebacteriaceae*, *Bacillus* spp., and *Enterococcus* spp.;
7. non-bacterial pathogens (yeasts, *Prototheca* spp.);
8. mixed infection (pathogens from two different categories).

While the IMI cure for each quarter was still determined for each single pathogen, the aforementioned pathogen groups were used in the statistical model to evaluate the influence of different pathogens or pathogen groups on the chance of cure during the DP. Further explanatory variables were the use of ITS at DO (dichotome), categorical SCC at DO (<100,000, 100,000–199,999, 200,000–299,999, 300,000–399,999, 400,000–999,999, >1,000,000 cells/mL); season of DO (December–February, March–May, June–August, September–November), categorical milk yield at DO (<10 kg, 10–14.9 kg, 15–20 kg, >20 kg), number of lactation (1, 2, 3, >3), BCS at DO (≤2.5, ≥3.75, 2.51–3.74), the change of BCS during the DP (<−0.5, −0.5–0.5, >0.5), and the categorical length of the DP (5–29 d, 30–49 d, 40–69 d, 70–89 d, >89 d). Categories were formed taking previous studies and commonly used methods into account. For example, for SCC, 100,000 and 200,000 cells/mL are thresholds often used by farmers in Germany and also described as reliable thresholds for identifying infected quarters at DO in previous studies [13,15], while others also investigated higher SCC for their influence on DP cure [29,42]. Since there was a wide range from very low to very high SCC measurements, we also added additional categories to account for the extremely high SCC and to enable more detailed results. As for the DP length, the categories were chosen to evaluate DP with common length as well as slightly and much longer DP. BCS categories were built to account for very low and very high BCS measurements at DO. Since BCS at DO might vary with DP length (e.g., cows with lower BCS being dried off earlier) and we included a wide range of DP length, a slightly wider range was set for the BCS thresholds than might be expected for recommended BCS at DO. Furthermore, the BCS change within the DP was included, since previous research described it to be more important for the *p.p*. udder health than the BCS at DO [30]. To adjust for different dry cow AB, their influence on DP cure was investigated in a reduced model including only quarters dried off with AB preparations. Therefore, the AB were grouped into categories according to their active substances, with single penicillin, penicillin and aminoglycosides, and cephalosporins being the three groups left in the final dataset (Table 5). The variables farm, cow, quarter, year of DO, pathogen category, DCT, season of DO, use of ITS, and length of DP were significant in the univariable analysis (*p* ≤ 0.20) and were therefore further investigated in the multivariable model. These variables had been tested for multicollinearity before inclusion in the multivariable model, although no high correlation (>0.7) was found between the variables. Hence all of the significant variables in the univariable model were tested in the multivariable analysis. 

The multivariable analysis was performed using a backward elimination procedure. After each run, the variable with the highest *p*-value was excluded from the model until all variables had *p* ≤ 0.05. Confounding was monitored by the change in the coefficient of a variable after removing another variable. If the change of the estimates exceeded 25% or 0.1 when the value of the estimate was between −0.4 and 0.4, the removed variable was considered a potential confounder and was re-entered in the model. 

In the final model, all possible two-way interactions were tested. With the pathogen-DCT interaction being kept in the model, the fix effects used in the model for the final analysis were the pathogen category at DO, the season of DO, the combination of DCT and pathogen category at DO as well as the DCT alone for being part of the interaction-term. Farm, cow, and year of DO were included as random effects. The robustness of the final model was checked by carrying out the same procedure with forward selection. The residuals of the final model were tested for normality and the coefficient of determination (r^2^) was calculated to measure the proportion of variance explained by the model. Statistical significance was set at a *p* value of <0.05 in the final model. Finally, estimated means were calculated for the explanatory variables included in the model.

## 5. Conclusions

Quarters with Gram-positive IMI, except NAS and *S. aureus*, were more likely to benefit from AB DCT compared to IMI caused by Gram-negative bacteria. An overall difference of 9% was seen in the cure rates between AB and non-AB treatment. Farm, cow, and season of DO also have a significant influence on the DP cure. Our findings indicate that the absolute effect of AB DCT depends on the pathogen causing the infection. To prevent AB resistance, a further reduction in AB treatments at DO might be possible with suitable selection protocols. Further research is needed to investigate the role of pathogens at the species level and the active compounds of dry cow AB. Additionally, DCT protocols considering IMI cure on the pathogen level need to be evaluated in future studies. 

## Figures and Tables

**Table 1:** Descriptive results for cow characteristics on quarter level for all quarters infected at DO^4^ and sorted by treatment group.

	Infected ^1^	Antibiotic Dry Cow Treatment				Non-Antibiotic Dry Cow Treatment			
	Mean (Median)	Mean (Median)	Min	Max	n ^2^	Mean (Median)	Min	Max	n
SCC ×1000 cells/mL ^3^	546 (197)	625 (253)	1	11,144	1037	502 (173)	1	51,352	1888
milk yield at DO ^4^	10.7 (10.0)	11.29 (11.0)	1	30.1	1025	10.5 (10.0)	0.2	27.8	1893
BCS ^5^	3.41 (3.5)	3.48 (3.50)	1.75	5.0	994	3.36 (3.25)	2.0	5.0	1610
parity	3.7 (3.0)	3.9 (3.0)	1	10	1010	3.6 (3.0)	1	19	1799
DP length (d) ^6^	57.1 (54.0)	58.1 (53.0)	15	149	1058	56.6 (54.0)	17	132	1929

^1^ all infected quarters matching the inclusion criteria; ^2^ number of quarters for which the variable was reported; ^3^ somatic cell count at dry off (×1000 cells/mL); ^4^ dry off; ^5^ Body Condition Score; ^6^ dry period length in days.

**Table 2:** Number of quarters infected at DO ^1^ and cured during the dry period in each DCT ^2^ group (AB ^3^ and non-AB) for all pathogen categories.

Pathogen Category	Infected at DO			Cure		
	Total n	AB n (%) ^4^	Non AB n (%) ^4^	Total n (%) ^5^	AB n (%) ^6^	Non AB n (%) ^6^
Total	2987	1058 (35.42)	1929 (64.58)	2342 (78.41)	894 (84.50)	1448 (75.06)
*Staphylococcus aureus*	332	133 (40.06)	199 (59.94)	279 (84.04)	123 (92.48)	156 (78.39)
NAS ^7^	1094	377 (34.46)	717 (65.54)	781 (71.39)	294 (77.98)	487 (67.92)
streptococci	255	85 (33.33)	170 (66.67)	206 (80.78)	80 (94.12)	126 (74.12)
Coliforms	224	118 (52.68)	106 (47.32)	202 (90.18)	103 (87.29)	99 (93.40)
other Gram-negative bacteria	63	26 (41.27)	37 (58.73)	58 (92.06)	21 (80.77)	37 (100.00)
other Gram-positive bacteria	668	188 (28.14)	480 (71.86)	580 (86.83)	184 (97.87)	396 (82.50)
non-bacterial infections	26	5 (19.23)	21 (80.77)	26 (100.00)	5 (100.00)	21 (100.00)
mixed infection	325	126 (38.77)	199 (61.23)	210 (64.62)	84 (66.67)	126 (63.32)

^1^ dry-off; ^2^ dry cow treatment; ^3^ antibiotic; ^4^ percentage of quarters in each treatment group from all quarters infected with a pathogen from the same group at DO; ^5^ proportion of quarters cured during the dry period from all infected quarters at DO for each pathogen category; ^6^ proportion of quarters cured during the dry period in each treatment group for each pathogen category; ^7^ non-aureus staphylococci.

**Table 3:** Generalized linear mixed model for quarter level bacteriological cure during the DP ^1^.

Effect	Odds Ratio	SE ^2^	*t*-Value	*p*-Value	95% CI ^3^ for Odds Ratio	
					Lower	Upper
Intercept	2.296	0.2992	2.778	0.006	1.277	4.128
**pathogen category**						
*S. aureus* ^4^	4.711	0.4059	3.819	<0.001	2.126	10.442
NAS ^5^	1.589	0.2582	1.794	0.073	0.958	2.637
streptococci	9.229	0.5440	4.086	<0.001	3.176	26.814
Coliforms	3.527	0.3803	3.314	<0.001	1.673	7.433
other Gram-negative bacteria	2.339	0.5808	1.463	0.144	0.749	7.305
other Gram-positive bacteria	20.100	0.5583	5.375	<0.001	6.727	60.063
non-bacterial pathogens	2,490,297.566	1056.0896	0.014	0.989	0.000	0.000
mixed infections	Reference					
**season of DO ^6^**						
Winter	1.423	0.2123	1.663	0.097	0.939	2.158
Spring	0.696	0.1715	−2.116	0.034	0.497	0.974
Summer	0.911	0.1503	−0.617	0.537	0.679	1.224
Autumn	Reference					
**DCT ^7^**						
Non-AB ^8^	0.925	0.2872	−0.270	0.787	0.527	1.625
AB	Reference					
**Interaction of DCT and pathogen category**						
Non-AB DCT in combination with each pathogen category:						
*S. aureus*	0.453	0.4795	−1.653	0.098	0.177	1.159
NAS	0.720	0.3187	−1.030	0.303	0.385	1.345
streptococci	0.140	0.6002	−3.274	0.001	0.043	0.455
Coliforms	1.932	0.5772	1.141	0.254	0.623	5.991
other Gram-negative bacteria	1,311,544.105	378.3516	0.037	0.970	0.000	0.000
other Gram-positive bacteria	0.126	0.5976	−3.468	<0.001	0.039	4.06
non-bacterial pathogens	1.009	1174.0970	0.000	1.000	0.000	0.000
corresponding AB group ^9^	Reference					

^1^ dry period; ^2^ Standard error; ^3^ Confidence Interval; ^4^ *Staphylococcus aureus*; ^5^ non-aureus staphylococci; ^6^ dry off; ^7^ dry cow treatment; ^8^ antibiotic; ^9^ non-AB DCT was compared to AB DCT within each pathogen category, thus, the Reference to non-AB DCT for one pathogen category is the matching AB treatment group for the same pathogen category.

**Table 4:** Estimated means for the dry period cure of infected quarters treated with or without antibiotics at dry off for each pathogen group.

Pathogen Category	DCT ^1^	Mean	SE ^2^	95% CI ^3^		Difference in Cure
				Lower	Upper	
*Staphylococcus aureus*	non-AB ^4^	0.815	0.039	0.726	0.880	
	AB ^5^	0.913	0.031	0.831	0.958	0.098
NAS ^6^	non-AB	0.703	0.041	0.618	0.776	
	AB	0.781	0.040	0.693	0.848	0.078
streptococci	non-AB	0.728	0.052	0.616	0.817	
	AB	0.954	0.023	0.880	0.983	0.226
coliforms	non-AB	0.934	0.027	0.855	0.971	
	AB	0.888	0.037	0.793	0.942	−0.046
other Gram-negative bacteria	non-AB	1.000	5.955 × 10^−5^	0.000	1.000	
	AB	0.840	0.078	0.629	0.942	−0.16
other Gram-positive bacteria	non-AB	0.840	0.029	0.774	0.889	
	AB	0.978	0.012	0.939	0.992	0.138
non-bacterial pathogens	non-AB	1.000	9.860 × 10^−5^	0.000	1.000	
	AB	1.000	0.000	0.000	1.000	0
mixed infections	non-AB	0.674	0.053	0.564	0.768	
	AB	0.691	0.060	0.562	0.796	0.017

^1^ dry cow treatment; ^2^ Standard Error; ^3^ Confidence Interval; ^4^ non-antibiotic DCT; ^5^ antibiotic DCT; ^6^ non-aureus staphylococci.

**Table 5:** All AB ^1^ mentioned in the primary dataset for use at DO ^2^ and AB left in the final dataset (*), within categories.

AB ^1^ Categories	AB Used at DO on the Farms
betalactam AB ^3^	cloxacillin/cloxacillin-benzathin *
	oxacillin *
	benzylpenicillin-procain *
	ampicillin combined with cloxacillin
combined betalactam & aminoglycoside AB ^3^	framycetin sulfate, benethamin-penicillin and penethamathydroiodid *
	benzylpenicillin-procain, benzylpenicillin-potassium and neomycin sulfate *
	benzylpenicillin-procain, dihydrostreptomycin and nafcillin *
	benzylpenicillin-procain and neomycinsulfat
Cephalosporins ^3^	cefazolin *
	cefquionome *
	cefoperazon
others	Gentamicin
	lincomycin combined with neomycin

^1^ antibiotics; ^2^ dry-off; ^3^ Categories left in the final dataset, that were investigated for influence on dry period cure; * AB used for dry cow treatment that remained in the final dataset, representing AB dry cow treatment of the quarters analyzed in this study.

## Data Availability

The data presented in this study are available on request from the corresponding author. The data are not publicly available due to reasons of privacy.
